# Peripheral-Free Calibration Method for Redundant IMUs Based on Array-Based Consumer-Grade MEMS Information Fusion

**DOI:** 10.3390/mi13081214

**Published:** 2022-07-29

**Authors:** Siyuan Liang, Xiaochao Dong, Tianyu Guo, Feng Zhao, Yuhua Zhang

**Affiliations:** 1 School of Communication and Information Engineering, Xi’an University of Posts and Telecommunications, Xi’an 710119, China; telestorm@163.com (S.L.); gtyuuu@163.com (T.G.); hfengzhao@xupt.edu.cn (F.Z.); 2 School of Computer Science and Technology, Baoji College of Arts and Science, Baoji 721016, China; zyhbzhy@163.com

**Keywords:** MEMS array, fixed error, fusion-calibration algorithm, LM-calibration algorithm

## Abstract

The MEMS array-based inertial navigation module (M-IMU) reduces the measurement singularities of MEMS sensors by fusing multiple data processing to improve its navigation performance. However, there are still existing random and fixed errors in M-IMU navigation. The calibration method calibrates the fixed error parameters of M-IMU to further improve navigation accuracy. In this paper, we propose a low-cost and efficient calibration method to effectively estimate the fixed error parameters of M-IMU. Firstly, we manually rotate the M-IMU in multiple sets of different attitudes (stationary), then use the LM-calibration algorithm to optimize the cost function of the corresponding sensors in different intervals of the stationary-dynamic filter separation to obtain the fixed error parameters of MEMS, and finally, the global fixed error parameters of the M-IMU are calibrated by adaptive support fusion of the individual MEMS fixed error parameters based on the benchmark conversion. A comparison of the MEMS calibrated separately by the fusion-calibration algorithm and the LM-calibration algorithm verified that the calibrated MEMS array improved the measurement accuracy by about 10 db and reduced the dispersion of the output data by about 8 db compared to the individual MEMS in a multi-dimensional test environment, indicating the robustness and feasibility of the fusion calibration algorithm.

## 1. Introduction

### 1.1. Background

Inertial navigation technology [1] is one of the most important navigation technologies and is characterized by high stealth, strong navigation autonomy, wide navigation coverage, etc. Meanwhile, MEMS sensors are one of the most important inertial devices in inertial guidance systems, with small size, light weight, low power consumption, easy integration, and other characteristics [2], which can make inertial navigation modules more integrated and miniaturized. Therefore, inertial navigation systems based on MEMS IMU have been developing rapidly in recent years. However, the low accuracy of MEMS in inertial sensors and measurement errors, such as high noise, limit their development and application [3] in military fields such as aviation, aerospace, high-end UAVs, and precision-guided bombs; in addition, MEMS gyroscopes have integral drift [4], and their accumulated noise is large during long working hours. Based on the current processing technology and fabrication process, it is difficult to rapidly reduce the system noise [5] of MEMS in a short period of time and improve the accuracy of individual MEMS [6], so forming MEMS arrays out of individual MEMS and using the redundancy [7] of MEMS arrays to further improve the accuracy of inertial systems have been a hot research topic in recent years.

Consumer-grade MEMS are often shipped from the factory without calibration for the fixed error parameters, which are caused by errors in the packaging process of the packaged IC [8] and non-orthogonality of the sensitive axes of the MEMS array, zero bias, and scale factor, etc. Therefore, this subject calibrates the individual MEMS [9] before the calibration of the MEMS array fusion [10], by compensating for the individual axis misalignment [11], zero bias, and scale factor of the MEMS, and then the MEMS array is analyzed for fixed errors. A reasonable MEMS calibration fixed error parameter [12] technique is a key process to reduce the system error.

### 1.2. Related Works

For the traditional calibration method, see John Baziw, Cornelius T. Leondes et al. [13,14] who proposed to use the data from navigation to calibrate the inertial guidance for fixed errors. This method requires accurate latitude and longitude information as an input to the system, collects the navigation north and east information at multiple positions, then models the position error information under the system and uses least squares to process it, and then uses an algorithm to calibrate the fixed error parameters. This method is limited to the number of calibration parameters and the accuracy of the parameters is poor. David Tedaldi and Alberto Pretto in [15] proposed a calibration scheme for multiple positions that guarantees system stability and is easy to implement, placing the sensor in a set of different static positions, accurately measuring the deviation of the sensor, and reliably and effectively estimating the fixed error parameters of the sensor. This method can accurately calibrate accelerometers, but for gyroscopes and magnetometers, the fixed error parameters cannot be accurately estimated. Zhang Xin et al. in [16] proposed a MEMS model based on nonlinear calibration factors and based on cone wobble motion and position oscillation as the excitation input to the MEMS, and then constructed a nonlinear IMU error model to calibrate the error parameters of multiple IMUs using a global weight function. Lukas Blocher, Wolfram Mayer in [17] used an extended Kalman filter with real-time error compensation for low-cost redundant MEMS for inertial devices to eliminate errors in angular random wander and bias instability, while using a rate table to fine-tune the system to compensate for fixed errors, and the compensated redundant MEMS was several times more accurate than a single MEMS. In [18], Hu Pei Da proposed a calibration method for inertial guidance system mounting errors based on attitude error models using a two-axis turntable device; the above three calibration methods are more accurate for specific parameters, but their calibration time is long and experimental equipment is expensive. JJan Rohac and Martin Sipos in [19], based on the calibration of accelerometers and gyroscopes, proposed to build the same error model but adopted a different calibration procedure, with the acceleration using a gravity-based position inversion method and the gyroscope using a calibration with measurements in the angular velocity and angular domains, with a reduction in deviation of about 6.28 db after calibration. The above calibration algorithms and testing techniques are often used to calibrate military-grade inertial equipment with high accuracy. Based on the irreplaceable role of consumer-grade inertial navigation equipment in civilian development, and with the improvement of electronic packaging technology, Feynman [20], the proposer of the MEMS concept and an internationally renowned physicist, once proposed that consumer-grade MEMS devices utilize no external device calibration algorithm [21] and filtering method [22]; processing and improving the accuracy of inertial navigation is an important topic in the development of MEMS navigation related technologies. Guided by this idea, the approach of multiple MEMS [23] undergoing fusion algorithms [24] to improve performance has received great attention in the study of inertial navigation, and, in turn, this technology has been given several names: batch MEMS [25], redundant MEMS [26], M-IMU [27], virtual MEMS [28], etc. All of which are largely based on the same principles and can be referred to as sensor array technology. John-Olof Nilsson in [29] obtained the error parameters of individual accelerations in an accelerometer array by collecting the static outputs of the accelerometer array for different positions using the traditional error IMU model with the maximum likelihood parameters using the Platonic solids on the positive 20 sides of the external device. In [30], Lu Jiazhen and Hu Maoqing derived measurement models by referring to the velocity information of satellite navigation, attitude information provided by the star sensor, and the measurement output of the redundant IMU, and calibrated the redundant IMU in orbit by setting three different levels of observation rotation sequences using Kalman filters, and then proposed a reliable calibration algorithm for the error parameters of the redundant IMU in orbit. The above method calibrates the multiple redundant IMUs one by one in a costly test instrument, but the IMUs in the array have different ranges, which in turn leads to large errors in the estimated scale factors.

The research work in the above literature has imperfections in both hardware and software algorithms: Hardware redundancy is scarce and MEMS sensors are single degree-of-freedom, resulting in less reliability and more measurement errors. The estimated error parameters solved by the algorithm are inaccurate and the calibration algorithm is complex, resulting in longer delays in output. The experimental steps for calibration are excessively cumbersome and do not accurately measure the error parameters in complex outdoor conditions.

### 1.3. Purpose of This Research

To address how to accurately eliminate randomness error parameters and calibrate fixed error parameters, we have chosen to calibrate individual MEMS in MEMS arrays based on consumer-grade experimental equipment, with redundant MEMS calibration as the main research component, so that their calibrated MEMS arrays can perform accurate inertial guidance without GPS [28]; specifically, the purpose of developing this work is twofold, as elaborated below.

(1) The IMU achieves peripheral-free real-time calibration [31] of a single IMU based on the elimination of its own random errors using ALLAN variance [32], which is achieved by calibrating a single IMU in real time in the attitude transformation of the inertial module, and recording the currently calibrated deterministic error parameters, and calculating the extremes of the deterministic error parameters of the single IMU through an iterative optimization algorithm. The iterative optimization algorithm calculates the extreme value of the deterministic error parameter of the individual IMU, thus obtaining a highly accurate individual IMU.
(2) Based on the calibrated individual IMU, the multi-sensor fusion algorithm [33,34,35] is referenced to obtain the fusion calibration algorithm proposed in this thesis, which is used to calculate the deterministic error parameters of the MEMS array, thus enabling pure inertial navigation without auxiliary equipment in complex outdoor environments in a short time.

## 2. Error Modelling of M-IMU and Cost Functions

### 2.1. Error Models for M-IMU

**Figure 1:**
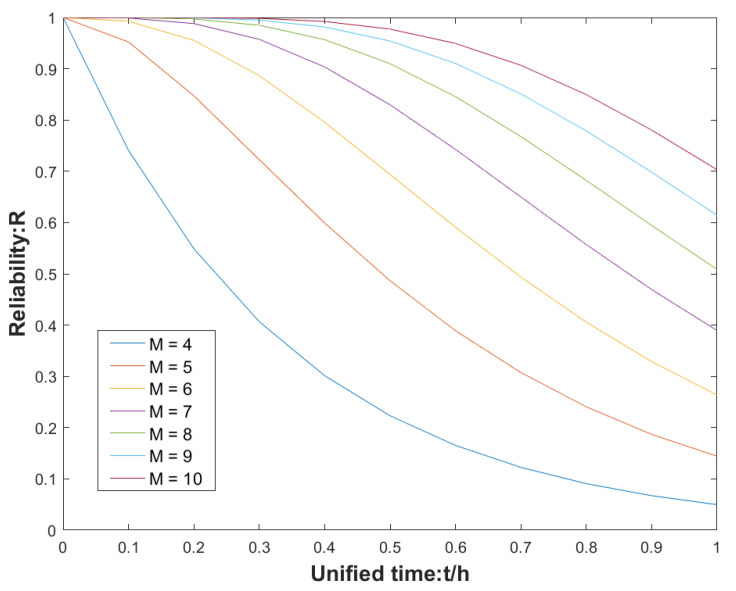
Reliability of a redundant system of three-axis inertial elements as a function of redundancy loss.

**Figure 2:**
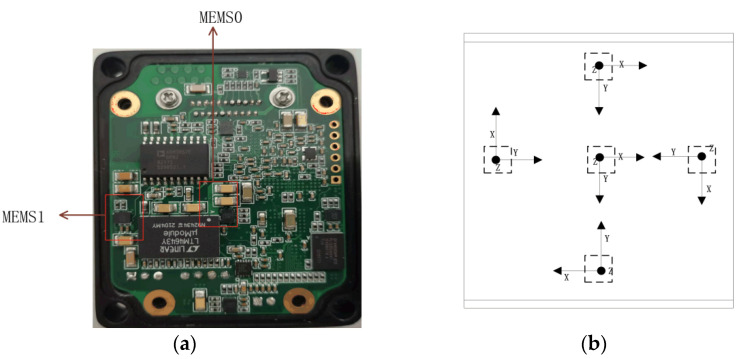
Spatial layout and orientation of the rotation axis of the MEMS array: (**a**) Spatial distribution of MEMS in the module; (**b**) Arrangement of sensitive axes in the module.

This study is aimed at M-IMU with 10 redundancies, which can reduce the measurement error of M-IMU while ensuring high reliability as shown in Figure 1 (M stands for redundancy of sensors). The M-IMU is arranged in front and reverse ways as shown in Figure 2, with the coordinates in the aerospace coordinate system of MEMS0 as the reference, and the layout of the MEMS space is considered under the premise that the volume constraint is within a certain range. The role of the layout should minimize the correlation between each MEMS and make the MEMS symmetrically and uniformly distributed, and thus the lower the correlation the better the fusion effect, in order to obtain better accuracy.

The fixed errors in the IMU come from three main components, including Bias and Noise, Scale errors, and Axis misalignments. The measurement model for accelerometers and gyroscopes can be expressed by the following equation.

(1) $$a_{i}^{B}=T_{i}^{a}K_{i}^{a}(a_{i}^{s}+b_{i}^{a}+v_{i}^{a})$$

(2) $$\begin{array}{l} \omega_{i}^{B}=T_{i}^{g}K_{i}^{g}(\omega_{i}^{s}+b_{i}^{g}+v_{i}^{g}+G_{g}a^{s})\\ i=1,2,\cdots ,10 \end{array}$$

where the superscript a represents the accelerometer, g represents the gyroscope, i represents the *i*-th sensor, and B represents the orthogonal reference coordinate system. S represents the transformation matrix of the axis deviations, K represents the scale factor, and $a_{i}^{s},\omega_{i}^{s}$ represents the true value of the output of the *i*-th accelerometer and gyroscope. b represents the zero bias, and v represents the random error.

$T_{i}^{a}$ is the mounting error matrix of the accelerometer, which shows the angular deviation of the non-orthogonal coordinate system mapped to the orthogonal coordinate system. For this MEMS, the error angle is relatively small and can be converted to a mounting error matrix in an orthogonal coordinate system. Meanwhile, the correction algorithm proposed in this topic converts $T_{i}^{a}$ into an upper triangular matrix, which in turn yields its orthogonal error angle, i.e.,

(3) $$a^{O}=T^{a}a^{S},T^{a}=\left[\begin{array}{ccc} 1 & -\alpha_{yz} & \alpha_{zy}\\ & 1 & -\alpha_{zx}\\ 0 & 0 & 1 \end{array}\right]$$

$a^{B}$ is the ratio in the orthogonal coordinate system represented, $a^{S}$ represents the ratio in the non-orthogonal coordinate system.

In the case of gyroscopes, reference should be made to the reference coordinate system of the accelerometer, as the calibrated gyroscope is based on a calibrated accelerometer so that installation errors causing system errors can be minimized. Its conversion to orthogonal coordinates under the installation of a non-orthogonal coordinate system becomes

(4) $$\omega^{O}=T^{g}\omega^{S},T^{g}=\left[\begin{array}{ccc} 1 & -\gamma_{yz} & \gamma_{zy}\\ \gamma_{xz} & 1 & -\gamma_{zx}\\ \gamma_{xy} & \gamma_{yx} & 1 \end{array}\right]$$

(5) $$\omega_{i}^{B}=T_{i}^{g}K_{i}^{g}(\omega_{i}^{s}+b_{i}^{g}+v_{i}^{g}+G_{g}a^{s})$$

(6) $$G_{g}=\left[\begin{array}{ccc} G_{1} & G_{2} & G_{3}\\ G_{4} & G_{5} & G_{6}\\ G_{7} & G_{8} & G_{9} \end{array}\right]$$

The measurement of the consumer-grade MEMS gyroscopes is based on the Coriolis force with ADC conversion to obtain the angular velocity. The output of the accelerometer affects the oscillation frequency of the MEMS and therefore the output of the gyroscope. Therefore, $G_{g}$ (a 3 × 3 bias matrix related to the gravitational acceleration *g*) is introduced to eliminate the effect of harmful accelerations.

### 2.2. Constructing Cost Functions for Accelerometers

The cost function is a convex function constructed by least squares, which in turn minimizes the cost function for a time window of $t_{w}$. The set of parameters to be solved for the accelerometer $\theta_{i}^{acc}=\left[\alpha_{yz},\alpha_{zy},\alpha_{zx},k_{x}^{a},k_{y}^{a},k_{z}^{a},b_{x}^{a},b_{y}^{a},b_{z}^{a}\right]$. Therefore, based on the traditional multi-position method with the addition of the rotation of the array MEMS, the output ratio of the accelerometer at each static interval of time is the average output ratio for that time period, and the cost function $L_{a}(\theta_{i}^{acc})$ for the parameters to be found in the accelerometer is constructed on the basis of the average ratio.

(7) $$a_{i}^{B}=h(a_{i}^{s},\theta_{i}^{acc})=T_{i}^{a}K_{i}^{a}(a_{i}^{s}+b_{i}^{a}+v_{i}^{a})$$

(8) $$L_{a}(\theta_{i}^{acc})=\frac{1}{2}{\sum_{k=1}^{M}\left(|\left|h(a_{i}^{s},\theta_{i}^{acc})|-|g\right||\right)}^{2}$$

$a_{i}^{s}$ the representation is the mean ratio of sensor i in a static time window, while ensuring that the ratio data for acceleration i is collected from multiple positions, at different attitudes.

### 2.3. Constructing the Cost Function of the Gyroscope

#### 2.3.1. Constructing the Attitude Transformation Matrix

The calibration of the gyroscope is improved on the calibrated accelerometer output $a_{i}^{B}$ by using the attitude transition matrix of the dynamic positional transition between the (*k*−1)-th static moment and the *k*-th static moment, thus constructing the cost function of the gyroscope. The attitude transition matrix of the sensor is expressed in the form of a quaternion q. The differential equation for the quaternion describes the attitude kinematics of the quaternion, and considering that the inertial guidance device is attached to the carrier, it is deduced that the measured angular velocity of the gyro is the absolute angular velocity along the carrier coordinate system, which in turn can be used to construct the traditional quadratic differential equation form, see Equation (8). Using the fourth-order Longacurta method RK4n to solve the above differential equation for the quaternion yields $\left[q_{1},q_{2},q_{3},q_{4}\right]$, Δt for the time interval, and $\left[q_{1},q_{2},q_{3},q_{4}\right]$ to construct the attitude transition matrix for Equation (9).


(9) $$Q\omega_{i}^{s}=\left[\begin{array}{l} {\dot{q}}_{0}\\ {\dot{q}}_{1}\\ {\dot{q}}_{2}\\ {\dot{q}}_{3} \end{array}\right]=\left[\begin{array}{cccc} q_{0} & -q_{1} & -q_{2} & -q_{3}\\ q_{1} & q_{0} & -q_{3} & q_{2}\\ q_{2} & q_{3} & q_{0} & -q_{1}\\ q_{3} & -q_{2} & q_{1} & q_{0} \end{array}\right]\left[\begin{array}{c} 0\\ \omega_{i(x)}^{s}\\ \omega_{i(y)}^{s}\\ \omega_{i(z)}^{s} \end{array}\right]=\frac{1}{2}\left[\begin{array}{cccc} 0 & -\omega_{i(x)}^{s} & -\omega_{i(y)}^{s} & -\omega_{i(z)}^{s}\\ \omega_{i(x)}^{s} & 0 & \omega_{i(z)}^{s} & -\omega_{i(y)}^{s}\\ \omega_{i(y)}^{s} & -\omega_{i(z)}^{s} & 0 & \omega_{i(x)}^{s}\\ \omega_{i(z)}^{s} & \omega_{i(y)}^{s} & -\omega_{i(x)}^{s} & 0 \end{array}\right]\left[\begin{array}{l} q_{0}\\ q_{1}\\ q_{2}\\ q_{3} \end{array}\right]$$

(10) $$Q_{i+1}=Q_{i}+\frac{h}{6}(s_{1}+2s_{2}+2s_{3}+s_{4}),h=\Delta t$$

(11) $$s_{1}=\frac{1}{2}\Omega_{i}q_{i},s_{2}=\frac{1}{2}\Omega_{i+\frac{1}{2}\Delta t}(q_{i}+\frac{\Delta t}{2}s_{1}),s_{3}=\frac{1}{2}\Omega_{i+\frac{1}{2}\Delta t}(q_{i}+\frac{\Delta t}{2}s_{2}),s_{4}=\frac{1}{2}\Omega_{i+\Delta t}(q_{i}+\frac{\Delta t}{2}s_{3})$$

(12) $$R=\left[\begin{array}{ccc} 1-2q_{2}^{2}-2q_{3}^{2} & 2(q_{1}q_{2}-q_{0}q_{3}) & 2(q_{0}q_{2}+q_{1}q_{3})\\ 2(q_{1}q_{2}+q_{0}q_{3}) & 1-2q_{1}^{2}-2q_{3}^{2} & 2(q_{2}q_{3}-q_{0}q_{1})\\ 2(q_{1}q_{3}-q_{0}q_{2}) & 2(q_{2}q_{3}+q_{0}q_{1}) & 1-2q_{1}^{2}-2q_{2}^{2} \end{array}\right]$$

#### 2.3.2. The Cost Function of the Gyroscope

The unknown parameter for gyroscope calibration can be expressed as: $\theta_{i}^{gyr}=\left[\gamma_{yz},\gamma_{zy},\gamma_{xz},\gamma_{zx},\gamma_{xy},\gamma_{yx},s_{x}^{g},s_{y}^{g},s_{z}^{g}\right]$, when the sensor moves, the gyroscope records the change in attitude of the gravity vector from the output of the calibrated accelerometer with the change in attitude vector as the sensor moves.

The subject defines the integration function ${\mathbb{R}}_{i}$ which takes as input the angular velocity of the gyroscope output $\omega_{i}^{s}$ for the *i*-th sensor with a total of M dynamic states and the initial gravity value $a_{init(K)}^{B}$ from the calibrated accelerometer and returns the final gravity value $u_{g,k}$ calculated from the quaternion conversion matrix (see Equation (11), R), which is also calculated for the (*K*−1)-th and *K*-th static intervals.

(13) $$u_{g,k}=\mathbb{R}[R_{k-1},\omega_{i}^{s},a_{i(K-1)}^{B}]=R_{k-1}\cdot a_{i(K-1)}^{B}$$

(14) $$L_{g}(\theta_{i}^{gyr})=\frac{1}{2}\sum_{k=1}^{M}\left(|\left|a_{i(k)}^{B}|-|u_{g,k}\right|\right|{)}^{2}$$

### 2.4. Optimization Algorithm for Minimizing the Cost Function

The LM (Levenberg-Marquarat) algorithm solves the nonlinear least squares extremum problem of the cost function (Equations (7) and (13)). The value of the objective function is required to decrease. If the decrease of the cost function satisfies the condition of decreasing sensor error, it means that the current iterative process is reliable, then continue to iterate and continue to calculate the optimal error parameter of the cost function. If the cost function The increase of the value, that is, the increase of the error, reduces the trust region range and recalculates, so the LM algorithm combines the advantages of the gradient descent method and the Gaussian descent method, and the LM algorithm adds an adjustment factor. Small increases the reliability of the data, making the entire formula close to the Gauss Newton method; when the convex function declines too slowly, it is used larger, the overall formula is close to the gradient method, the data convergence speed is accelerated, and the running algorithm time is reduced. The LM algorithm is shown in the following formula:

(15) L(x+Δx)≈L(x)+J(x)Δx

(16) $$\Delta x={\left(H+\alpha D\right)}^{-1}g$$

(17) $$H=J{\left(x\right)}^{T}J(x)$$

(18) $$g=-J{\left(x\right)}^{T}L(x)$$

L is the cost function shown in Equations (7) and (13); Δx represents the increment; H is the Hessian array, represented here by the J(x) Jacobi matrix; D is the transformation matrix of the increment, and λ is the trust domain radius or damping factor.

The incremental function of the above equation is brought into the iterative equation of the LM algorithm, and the error parameters optimized for the MEMS in the current physical environment are determined by iterative referencing of *K*-th static positions.

### 2.5. Algorithm for Fusing Calibration Parameters

The fixed error of MEMS is often linear at narrow measurement ranges and non-linear at wide measurement ranges, while the error parameters of individual MEMS calibrated based on LM algorithms are often accompanied by parameter singularities due to changes in temperature, air pressure, and other real-world conditions. In order to increase the attitude conversion range and to avoid error parameter singularities, this project selects an array of multiple MEMS and applies an improved fusion algorithm to enable the inertial guidance system to increase the measurement range while using the adaptive support of the sensors to weight and fuse the error parameters into a “high accuracy virtual inertial guidance” all the way to circumvent the singularity of the measured values. The above arrayed inertial guidance system has five sensors on one side, a central MEMS, and four MEMS arranged in a rectangular pattern. The rectangular MEMS are converted according to the coordinate system conversion equation as shown in the following Equations (18) and (19) and the center MEMS coordinate system base center alignment. The application of the rectangular layout of the MEMS array can reduce temperature drift, while using the coordinate conversion equation to convert the sensor coaxial isotropic, and then the MEMS are weighted and fused into a set of “high precision virtual inertial guides” using a “virtual fusion algorithm”. The “virtual determination of error parameters” for this “high precision virtual fusion inertial guide” directly affects the accuracy of the MEMS array output data, so the “virtual high precision inertial guide”, which can accurately calibrate the MEMS array on the basis of the LM-based calibration algorithm that can accurately calibrate the error, becomes another research point of this paper.

For this project, the spatial layout of the MEMS array is orthogonal, and is based on the correlation between redundant sensors of the same category. The conversion matrix (Equations (19) and (20)) is used to perform a coordinate transformation with the central MEMS0 as the reference, then the fusion-calibration algorithm is used to calibrate the MEMS array fusion weighted into a “high precision virtual inertial guidance“, deterministic error parameters, and random error parameters of MEMS0.

(19) $$R_{0}=\left[\begin{array}{ccc} 1 & 0 & 0\\ 0 & 1 & 0\\ 0 & 0 & 1 \end{array}\right]\;R_{1}=\left[\begin{array}{ccc} 0 & 1 & 0\\ -1 & 0 & 0\\ 0 & 0 & 1 \end{array}\right]\;R_{2}=\left[\begin{array}{ccc} -1 & 0 & 0\\ 0 & -1 & 0\\ 0 & 0 & 1 \end{array}\right]\;R_{3}=\left[\begin{array}{ccc} 0 & -1 & 0\\ 1 & 0 & 0\\ 0 & 0 & 1 \end{array}\right]\;R_{4}=\left[\begin{array}{ccc} 1 & 0 & 0\\ 0 & 1 & 0\\ 0 & 0 & 1 \end{array}\right]$$

(20) $$R_{0^{\prime }}=\left[\begin{array}{ccc} 1 & 0 & 0\\ 0 & 1 & 0\\ 0 & 0 & -1 \end{array}\right]\;R_{1^{\prime }}=\left[\begin{array}{ccc} 0 & 1 & 0\\ -1 & 0 & 0\\ 0 & 0 & -1 \end{array}\right]\;R_{2^{\prime }}=\left[\begin{array}{ccc} -1 & 0 & 0\\ 0 & -1 & 0\\ 0 & 0 & -1 \end{array}\right]\;R_{3^{\prime }}=\left[\begin{array}{ccc} 0 & -1 & 0\\ 1 & 0 & 0\\ 0 & 0 & -1 \end{array}\right]\;R_{4^{\prime }}=\left[\begin{array}{ccc} 1 & 0 & 0\\ 0 & 1 & 0\\ 0 & 0 & -1 \end{array}\right]$$

**Figure 3:**
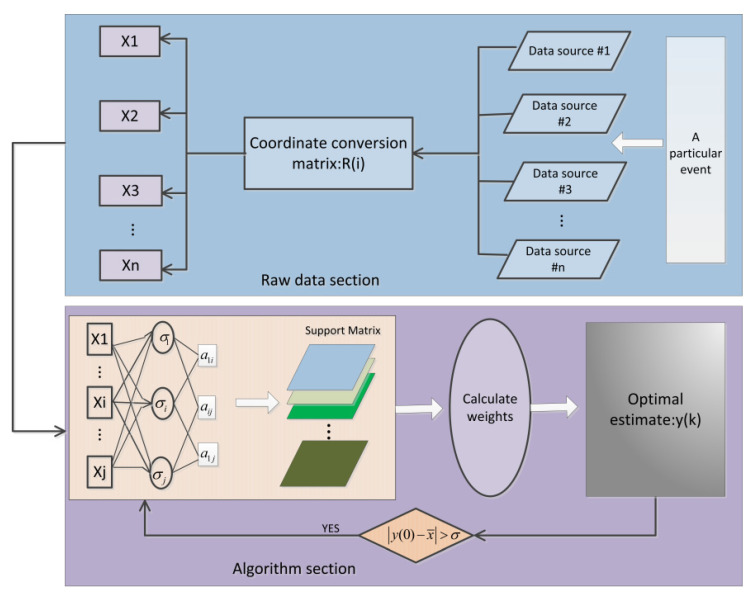
Flow chart of the algorithm for fusing calibration parameters.

The fusion-calibration method with improved support proposed in this topic is shown in Figure 3. Each sensor is fused with each parameter independently based on the fixed error parameters of the conversion matrix. If there is still a large error between the data after the initial fusion of the sensor and the actual measured value, the initial fused data need to be judged by the error threshold ψ. If it is less than the threshold, the fusion is successful; otherwise, the initial fused data are fused again with the sensor parameters.

(21) $$x_{i}=f(R_{i}\theta_{i}^{sen})$$

(22) $$\psi =\max\left|x_{i}-x_{j}\right|$$

(23) $$\eta =\max(x_{1}^{k}-x_{i})$$

$x_{i},x_{j}$ represent the data of a fixed error parameter of sensor *i* and sensor *j*, respectively; $R_{i}$ represents the conversion matrix of sensor *i*; f represents the sensor error parameter $R_{i}\theta_{i}^{sen}$ with respect to the separation function, after the separation conversion; sen represents the accelerometer or gyroscope; ψ represents the maximum threshold value between sensors; and η represents the maximum threshold value of the result of the initial fusion $x_{1}^{k}$ with respect to a fixed error parameter of sensor *i*.

An improved exponentially decaying support function to describe the support between individual sensors is expressed as follows.

(24) $$\sup_{ij}=a_{ij}=\exp(-\beta {\left(x_{i}-x_{j}\right)}^{2}),i\neq j(1,2,\cdots 10);\eta \leq \psi$$

(25) $$\sup_{im}=a_{im}=\exp(-\beta {\left(x_{i}-x_{m}\right)}^{2}),x_{m}(x_{j},x_{1}^{k});\eta >\psi$$

(26) $$s_{i}=\frac{1}{1+\sigma_{i}}$$

(27) $$\sigma_{i}=\frac{\sum_{i=1}^{n}\left((x_{i}-\bar{x}\right){)}^{2}}{n},(n=10,\eta \leq \psi );(n=11,\eta >\psi )$$

(28) $$\beta_{a}=\sqrt{s_{i}s_{j}},i\neq j(1,2,\cdots 10);\eta \leq \psi$$

(29) $$\beta_{b}=\sqrt{s_{i}s_{m}},i\neq m(x_{j},x_{1}^{k});\eta >\psi$$

The above equation uses $\sigma_{i}$ to represent the variance of the output data of sensor *i* per unit time; $\sup_{ij}$, $\sup_{im}$ represent the support of the observations of sensor *i* and sensor *j*; $\bar{x}$ represents the mean value of sensor *i* per unit time; $\beta_{a}$, $\beta_{b}$ are adjustable parameters, which in turn modulate the metric scale; when η≤ψ, Equations (24) and (26)–(28) form the adaptive support function of sensor *i* and sensor *j*; when η>ψ, Equations (25)–(27) and (29) form the adaptive support function of sensor *i*, sensor *j* and the first fusion result $x_{1}^{k}$.

The matrix of mutual support between the sensors is:

(30) $$A=\left[\begin{array}{cccccc} a_{11} & a_{12} & \cdots & a_{1j} & \cdots & a_{1n}\\ a_{21} & a_{22} & \cdots & a_{2j} & \cdots & a_{2n}\\ & \vdots & & \ddots & & \vdots \\ a_{i1} & a_{i2} & \cdots & a_{\mathrm{ij}} & \cdots & a_{in}\\ \vdots & \vdots & \cdots & \vdots & \vdots & \vdots \\ a_{n1} & a_{12} & \cdots & a_{\mathrm{nj}} & \cdots & a_{nn} \end{array}\right]$$

The normalization of the total support yields a consistency measure function for sensor *i* as:

(31) $$w_{i}(k)=\frac{sum(i)}{n-1},i=1,2\cdots 10;0<w_{i}\leq 1$$

The consistency mean of the *i*-th sensor at moment *K* of unit time is expressed as:

(32) $$\bar{w_{i}(k)}=\left\{\begin{array}{c} w_{i}(1),k=1\\ \frac{k-1}{k}\bar{w_{i-1}(k)}+\frac{1}{k}w_{i}(k),k>1 \end{array}\right.$$

The observed consistency variance of the *i*-th sensor at moment *K* of unit time is:

(33) $$\lambda_{i}^{2}(k)=\left\{\begin{array}{c} 0,k=1\\ \frac{k-1}{k}\lambda_{i}^{2}(k-1)+\frac{1}{k}{\left[\bar{w_{i}(k)}-w_{i}(k)\right]}^{2},k>1 \end{array}\right.$$

In the actual fusion process, full use should be made of observations from sensors with large consistency mean values and small consistency variance at the same time, so that the correlation weight of the ith sensor observation at moment *K* is:

(34) $$\mu_{i}(k)=\frac{\bar{w_{i}(k)}}{\alpha +a\lambda_{i}^{2}(k)},i=1,2\cdots n$$

In the above equation, α is the noise constant of the system observations; a is an adjustable parameter disturbed by the environment, by adjusting this parameter the effect of $\lambda_{i}^{2}(k)$ on the weighting parameters can be changed. The final fusion estimates obtained are:

(35) $$x_{m}^{k}=\frac{\sum_{i=1}^{n}\left[\mu_{i}(k)x_{i}(k)\right]}{\sum_{i=1}^{n}\mu_{i}(k)},i=1,2\cdots n=10;m=1\;\mathrm{or}\;2$$

The practical superiority of this fusion calibration algorithm compared to other calibration algorithms is shown in Table 1. The measurement variance of this fusion calibration algorithm is influenced by a variety of factors, including both the effect of noise in the sensor system and environmental disturbances, etc. This adaptive weighted fusion algorithm ensures the reliability of the sensor measurements and minimizes the total variance of a fixed error parameter obtained after fusion.

## 3. MEMS Array Calibration Experiments

### 3.1. Calibration Experimental Procedure

#### 3.1.1. Static and Dynamic Filters

As mentioned earlier, the raw data stream is divided into two forms: smooth and moving. The accuracy of the calibration then depends heavily on the reliability of classifying the static and dynamic, using the static interval to calibrate the accelerometer, using the motion interval between the *K*-th static interval and the (*K*−1)-th static interval to calibrate the Gyroscope, while the output data from the calibrated accelerometer at the (*k*−1)-th moment are used as the standard quantity in Equation (13) for comparison, and thus the gyroscope error parameters are calibrated to the optimum value.

The static and dynamic filter is constructed based on a band-pass filter, with the variance Equation (25) of the data in the time period with $T_{init}$, constructed as a comparison quantity, judged on the following basis.

(36) $$\begin{array}{l} \varsigma (t_{init})=\frac{\sum_{i=1}^{m}\sqrt{{\left[{\mathrm{var}}_{tw}(a_{x}^{t})\right]}^{2}+{\left[{\mathrm{var}}_{tw}(a_{y}^{t})\right]}^{2}+{\left[{\mathrm{var}}_{tw}(a_{z}^{t})\right]}^{2}}}{m}\\ i=1,2,\cdots m \end{array}$$

**Figure 4:**
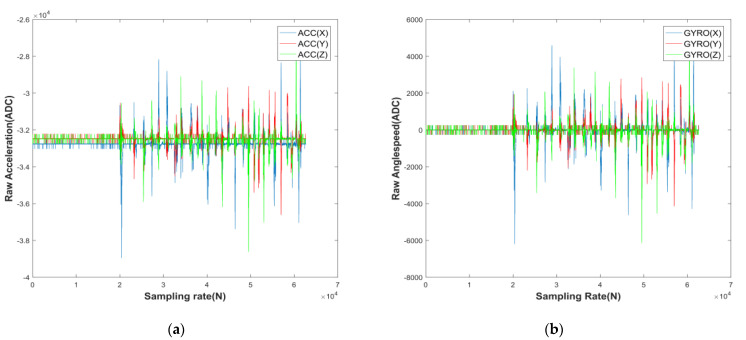
Static and dynamic distribution of accelerometers and gyroscopes: (**a**) dynamic distribution of accelerometers; (**b**) dynamic distribution of accelerometers.

The above equation ${\mathrm{var}}_{tw}(a^{t})$ represents the variance of the acceleration $a^{t}$ over the time period $t_{w}$. To determine the motion of the MEMS array, simply compare $\varsigma (t_{w})$ with the threshold $\varsigma (t_{init})$, larger is motion and the opposite is static, while taking $t_{w}=2s$, $t_{init}=50s$; Figure 4 below reflects the static and dynamic distribution of the entire sensor pose transition.

#### 3.1.2. Calibration Procedure

The calibration algorithm is composed of a data acquisition part and iterative optimization of the parameters part. In the data acquisition phase, the raw data of the IMU are collected from different sensor MEMS_i_; this inertial guidance module is calibrated by placing it in M different positions, and by placing it stably in each position attitude for at least $t_{w}$ s, also determines the time $t_{init}=50s$ of the first pose placement. The calibration intervals in the optimal parameters section are determined by manually adjusting the attitude transitions of the sensors, using static positional placement and dynamic attitude transitions to calibrate the gyroscopes and accelerometers in the MEMS, respectively.

**Figure 5:**
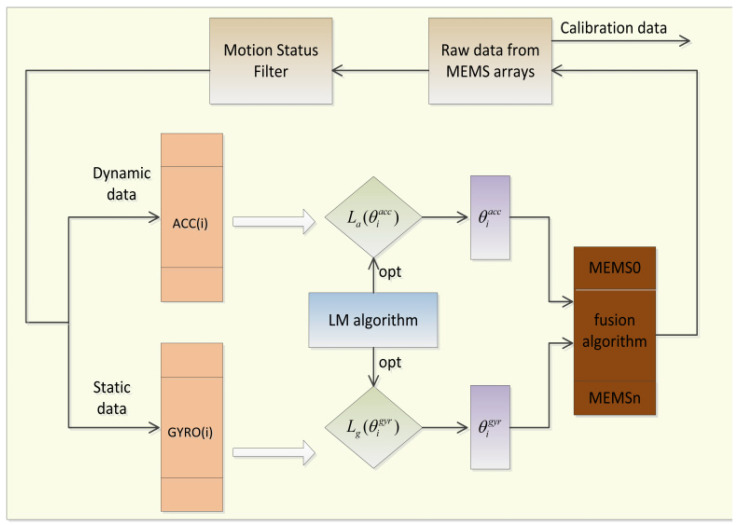
Flow chart of the overall calibration.

Based on the fixed error parameters of the calibrated individual MEMS, the subject proposes a fusion calibration algorithm for the convergence of M-IMU. Firstly, we exclude the case of random wandering in the random error, when the IMU will have an absolute error calibration parameter, and secondly, the optimization of the minimum value error will occur when optimizing the cost function in the calibration algorithm. If the optimal values found at $\theta^{acc}$ and $\theta^{gyro}$ satisfy the smallest subcost function value, the estimate is close to the absolute calibration parameter. In order to avoid being trapped in a local optimum, an improved fusion function is introduced to pinpoint the global optimum based on the adaptive weights, and the entire calibration experiment flow is detailed in Figure 5.

### 3.2. Calibration of MEMS Data Parameters for Real Experiments

**Figure 6:**
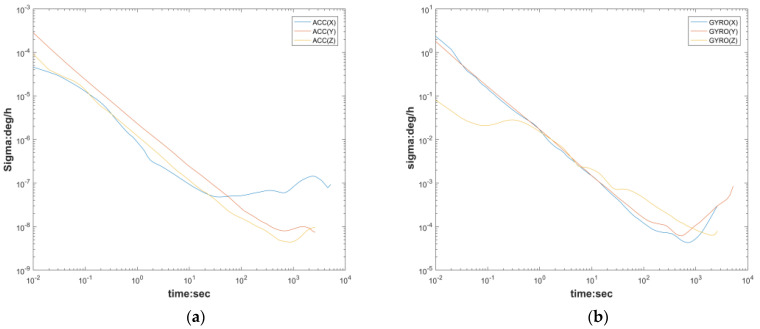
Allen variogram for accelerometer (MEMS0) and gyroscope: (**a**) Accelerometer; (**b**) gyroscope.

The experimental data are divided into two data streams according to the static and dynamic filter: the first group is the attitude stationary data of the MEMS array used to calibrate the accelerometer, and the second group is the attitude rotational data of the MEMS array used to calibrate the gyroscope. The random error of the eliminated MEMS array is based on MEMS0, and the random error parameters are analyzed using ALLAN, as shown in Figure 6 and Table 2 below, while the random error parameters and the fixed error parameters T,K,b form a model, as shown in Equations (1) and (2), and the above model is transformed into the following equation:

(37) $$a_{i}^{s}={\left(T_{i}^{a}K_{i}^{a}\right)}^{-1}a_{i}^{B}-b_{i}^{a}-v_{i}^{a}$$

(38) $$\omega_{i}^{s}={\left(T_{i}^{g}K_{i}^{g}\right)}^{-1}\omega_{i}^{s}-b_{i}^{g}-v_{i}^{g}-G_{g}a^{s}$$

The above Equations (31) and (32) separate the observed and actual quantities and then use the actual experimental data for the calibration of the MEMS array. So far, there is no consensus data set for the calibration of the IMU, and we do not use external tools with high accuracy such as tri-axis turntables or star-sensors in order to better match the actual environment, e.g., rainforest wetlands, bad weather, etc. To obtain accurate parameters, experiments in Section 4 are needed to demonstrate the accuracy of the algorithm in calibrating the sensors.

The experiment was conducted using a six-axis IMU sensor from ST, LSM6DS0, which uses a gyroscope with an actual range of ±500 dps (degree per second) and an accelerometer with an actual range of ±4 g. The IMU sensor was manually converted to attitude and about 300 (30 × 10) sets of data were collected under the conditions for calibration, and the calibrated results are shown in Table 3.

**Table 2:** Allen parameters for the accelerometer (MEMS0) and gyroscope.

Parameters	Accelerometers			Gyroscope		
	White Noise	Bias Instability	Random Walk	White Noise	Bias Instability	Random Walk
*X*-axis	0.0012	1.5702 × 10^−5^	3.3759 × 10^−4^	0.1219	5.6467 × 10^−4^	0.0119
*Y*-axis	0.0015	5.0196 × 10^−6^	1.3451 × 10^−4^	0.1219	5.6467 × 10^−4^	0.0119
*Z*-axis	0.0011	3.5621 × 10^−6^	1.0334 × 10^−4^	0.1526	0.6689	0.2182

Table 3 above calibrates all the fixed error parameters of the MEMS*_i_* (*i* = 1, 2,…, 10) in the MEMS array. The error parameter estimates vary for each MEMS calibrated due to the different axes of each MEMS as in Figure 1, and, also, in different calibration environments, air pressure, current strength, and other actual environments can affect the parameters leading to data singularities Therefore, according to the fusion calibration algorithm Equations (18) and (19), the coordinates are converted to the coordinate system of the reference MEMS0, and then according to the adaptive support Equation (17), the fused fixed error parameters are shown in Table 4, Table 5 and Table 6, which become the global optimal values, and the M-IMU system compensates for the above-fused calibration error parameters to make the MEMS0 an inertial device that can perform accurate autonomous navigation at a certain time.

## 4. Validation and Discussion

The fusion-calibration algorithm for multi-sensor data combines the measurement parameters from each sensor to obtain more accurate, stable, and reliable global parameters, thus minimizing the singularities [16] in the multi-sensor measurement data. The global variables obtained in Table 4, Table 5 and Table 6 above must be evaluated against the following metrics to verify the authenticity of the fusion- calibration algorithm:

(1) Static measurements: compare the static output of the original data [36], the static output after calibration by the LM-calibration algorithm, the static output of the fusion-calibration algorithm, compare the estimated value of the output data with the measured value, and simultaneously measure the RMSE of the above three sets of data per unit of time.
(2) Dynamic measurements: for accelerometers, uniform acceleration motion in the X-O-Y plane at a certain acceleration to measure the accuracy of the acceleration; for gyroscopes, rotation at a fixed angular rate using a single axis turntable to compare the accuracy of the angular rate after LM-calibration, and then after fusion-calibration.
(3) Integrated measurement: the GPS prescribed path as a benchmark, the planned trajectory is in the width of 2 m, the total length of 4 km road vehicle travel route, the vehicle in the first 10 s uniform speed straight line walking, 10–100 s accelerated curve walking, after deceleration to the end of the actual environment there are trees, tall buildings shade, obstacles road stalls and other interference, in the vehicle speed and road conditions complex The GPS planned path, the inertial guidance path of LM calibration algorithm and the inertial guidance path of fusion calibration algorithm are observed in the situation. The path coincidence, planar displacement error and skyward displacement error of these three solutions are analysed.

### 4.1. Static Validation

**Figure 7:**
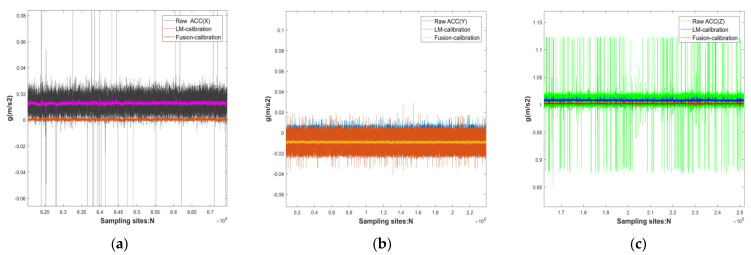
Comparison of original sensor, calibrated single MEMS, and fused calibrated MEMS in the MEMS0 accelerometer at standstill: (**a**) *X*-axis of the accelerometer; (**b**) *Y*-axis of the accelerometer; (**c**) *Z*-axis of the accelerometer.

**Figure 8:**
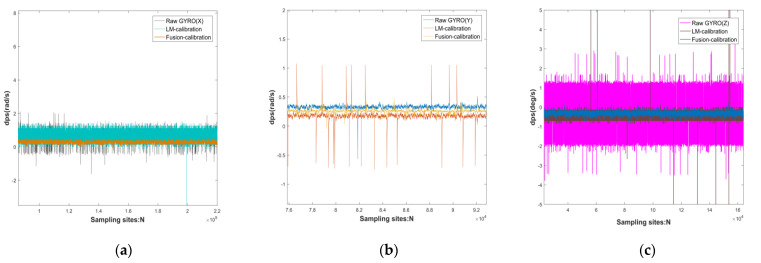
Comparison of original sensor, calibrated single MEMS, and fused calibrated MEMS in MEMS0 gyroscope at standstill: (**a**) *X*-axis of the gyroscope; (**b**) *Y*-axis of the gyroscope; (**c**) *Z*-axis of the gyroscope.

Data were collected for 30 min at rest, constant temperature, and pressure: raw accelerometer and gyroscope (MEMS0), accelerometer and gyroscope calibrated by the LM algorithm (MEMS0), and accelerometer and gyroscope calibrated by the fusion-calibration algorithm (MEMS0). The static errors of the current MEMS0 output data were then compared, while the obtained estimates were compared with the true values, see Figure 7 and Figure 8 and Table 7 and Table 8.

The reliability of the calibration fusion algorithm can be illustrated according to Figure 7 and Figure 8 and Table 7 and Table 8. For accelerometers: the data output from the acceleration-sensitive axes at rest is more accurate, but the singularity of the calibration error parameter still occurs. In the ideal case of an accelerometer in the gravity coordinate system with *X*-axis, *Y*-axis at 0 g, and accelerometer *Z*-axis at 1 g (g being the local gravitational acceleration), an individual MEMS calibrated accelerometer improves accuracy over the original accelerometer in the sensitive axis: 16.49 db, 1.10 db, 0.95 db, and its LM algorithm calibrated accelerometer improves stability over the original accelerometer in the *X*, *Y*, *Z* axis: 19 db, 8 db, 15 db, respectively; for gyroscope: at rest, the gyroscope is affected by the frequency of vibration, so the calibration of fixed error is often the key to reduce the initial attitude error and improve IMU navigation, the above LM algorithm calibrated gyroscope original gyroscope in the sensitive axis accuracy improvement: 1.41 db, 0.3 db, 0.52 db, its LM algorithm calibrated gyroscope improves stability over the original gyroscope in the *X*, *Y*, *Z* axis: 17.44 db, 8.96 db, 3.60 db, respectively.

The consumer-grade MEMS raw data have more wild values, and the LM-calibration algorithm enables the MEMS to increase the convergence of the data, but to avoid local optimum solutions for the MEMS array, the error parameters of the MEMS array are weighted and fused using the adaptive support of the calibration algorithm to make MEMS0 a set of high-precision inertial guides. For accelerometers: The fusion calibration algorithm improves the accuracy of the static raw data calibrated by the LM calibration algorithm: 11.46 db, 2.96 db, 6.13 db. The fusion algorithm improves the convergence of the accelerometers calibrated by the LM algorithm in the *X*, *Y*, *Z* axis: 7.69 db, 13.26 db, 5.12 db, respectively.

### 4.2. Dynamic Validation

**Figure 9:**
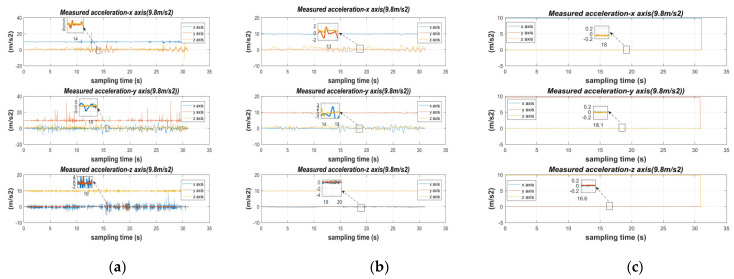
MEMS0 accelerometer in dynamic conditions: (**a**) raw sensor; (**b**) LM-calibrated sensor; (**c**) fused calibrated sensing.

**Figure 10:**
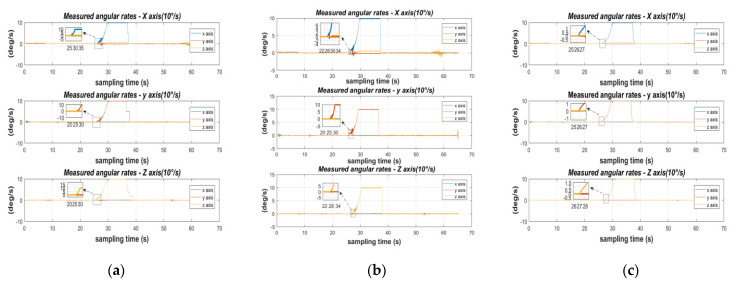
MEMS0 gyroscope in dynamic conditions: (**a**) raw sensor; (**b**) LM calibrated sensor; (**c**) fused calibrated sensing.

In a static state, the sensor is greatly affected by random errors, while ignoring the influence of the actual environment, such as the influence of dynamic errors such as the earth’s rotation and vibration frequency. At the same time, the quaternion transformation matrix of Equation (29) uses the angular velocity of the gyroscope as a parameter, and the acceleration and pose play an important role in the inertial navigation of the IMU. Therefore, dynamic verification has become the MEMS array of this topic as an important support in inertial navigation. The working environment of the dynamic verification is carried out under the experimental conditions of constant temperature and constant pressure. The accelerometer is tested with a constant local gravitational acceleration around the sensitive axis, and the gyroscope is tested with a fixed angular rate of 10 rad/s. For the comparison unit time: original data, data after calibration by LM algorithm, data after fusion calibration, and mean value and root mean square error of the above three data, see Figure 9 and Figure 10 and Table 9 and Table 10 for details:

The above acceleration calibration experiment is based on the perceived gravitational acceleration in the static interval as a known quantity, and the cost function is constructed from the known quantity, so it is important to verify the gravity vector and root mean square error of each axis, which can be obtained from Figure 9, after the LM-calibration algorithm increases the convergence of the original sensing, and after the fusion-calibration algorithm searches for the global optimal value, which, in turn, accurately calibrates the fixed error of MEMS0 parameters.

The experimental procedure for verification was as follows: the coordinate system of the MEMS array module was aligned with the local Cartesian coordinates coordinate system (ENU), and then the sensitive axes were aligned with the direction of the gravity vector, and the data from the three axes of the accelerometer were collected in this state for 30 s. The rotation around the sensitive axes in the table above: the acceleration accuracy of the sensor calibrated by the LM algorithm and the original sensor was improved: 0.02 db, 4.26 db, 1.76 db, and the stability of the data was improved: 6.01 db, 3.07 db, 1.18 db. The fusion calibration algorithm improves the accuracy of the sensor calibrated by the LM algorithm by 1.59 db, 4.33 db, 15.31 db and the stability of the data by a significant margin.

The dynamic verification process of the gyroscope is shown in Figure 10 above as follows: using a single axis turntable at 10 rad/s, the estimated value of the gyroscope is compared to the measured value. The initial stationary state is maintained for about 25 s and then a 10 s rotation around the sensitive axis is performed to collect data from the other axes. Finally, another 10 s of stationary is performed. Since the certainty of the dynamic attitude transformation of Equation (29) is concerned with the accuracy of the gyroscope calibration results, this above dynamic scheme is also based on the accuracy of the quaternion transformation matrix.

Table 10 above verifies that the gyroscopic accuracy of the sensor calibrated by the LM algorithm and the original sensor in a rotated state around the sensitive axis is improved: 1.90 db, 3.03 db, 3.10 db; its data stability is improved: 14.15 db, 15.21 db, 11.38 db. The fusion calibration algorithm improves the accuracy of the sensor calibrated by the fusion calibration algorithm over the LM algorithm: 4.71 db, 7.67 db, 9.20 db. 7.67 db, 9.20 db and its data stability improved: 6.31 db, 1.18 db, 10.18 db.

### 4.3. Integrated Validation

The above static and dynamic measurements were performed under more rational laboratory conditions, verifying that the individual sensors in the MEMS array have good estimates under laboratory conditions. However, M-IMUs are based on inertial navigation products in real-world navigation environments, and M-IMUs use attitude, heading, and odometry information in heading projections to project relative positions to the starting point, so accurate evaluation of the performance of calibrated and compensated M-IMUs in real-world dynamic navigation [37,38] will be the focus of this section.

**Figure 11:**
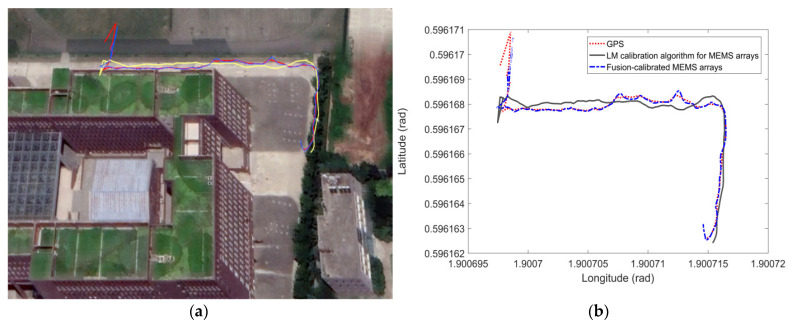
Trajectory in Google Earth 3D map and trajectory solved by MEMS array: (**a**) Actual environmental trajectory; (**b**) Planar trajectory.

The experimental procedure was chosen to plan the driving trajectory in full open-air conditions; the trajectory has site tall buildings, trees, obstacles barricades, and other complex terrain environments, see Figure 11. The verification experimental steps have been described in detail in the preamble of Section 4, and the GPS positioning results were compared with the trajectory solved by the MEMS array, the MEMS array calibrated by the LM-calibration algorithm, and the MEMS calibrated by the fusion-calibration algorithm. The RMSE of the horizontal position and the initial error misalignment angle error in the localization error are compared.

**Figure 12:**
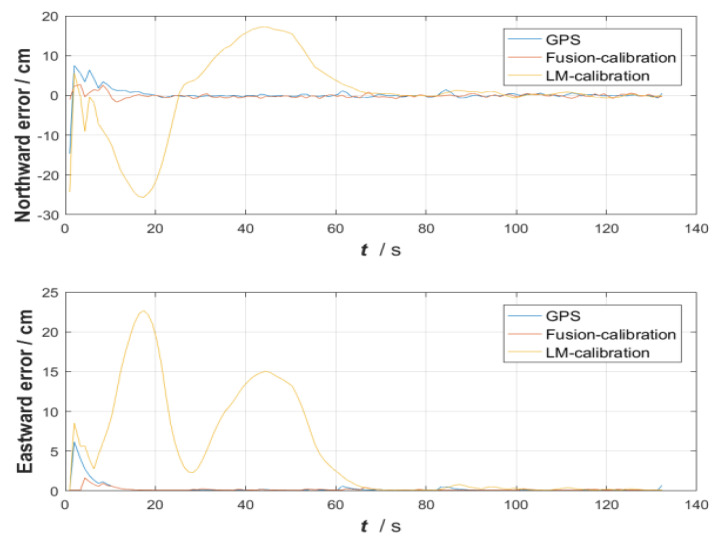
Vertical comparison error and horizontal comparison error.

The difference in effectiveness between the fusion-calibrated MEMS array and the LM-calibration algorithm MEMS in real-world inertial navigation is still relatively obvious. The vertical and horizontal errors in the navigation process were analyzed by comparing the results of the calibrated MEMS array with those of the individual MEMS, using GPS as a benchmark, and are shown in Figure 12. In the first 8 s of inertial navigation, the modules are coarsely aligned to the navigation coordinate system and then the individual MEMS are placed in the sun for the trajectory tour. In the first 50 s, the errors in the individual MEMS are greater due to poor calibration of the MEMS by the LM algorithm and the temperature drift of the modules caused by the sunlight. At 60–72 s during the process, the individual MEMS sensor modules are less affected by the temperature drift due to the shading of trees and buildings. The MEMS array was laid out as shown in Figure 1 to effectively suppress the effects of temperature drift, while the fusion calibration algorithm was used to accurately calibrate the fixed error parameters in the MEMS array, which in turn enables the inertial navigation of MEMS0 to be rapidly improved in a short period of time. The following analysis of the northward root mean square error and eastward root mean square error as well as the misalignment angle error after 3 s is shown in Table 11.

Table 11 above shows the root mean square errors of the calibrated MEMS array and calibrated MEMS in the north and east directions in the entire inertial navigation. The whole experiment of this verification is also planned in the “North–East” direction. It is shown in the table above that the convergence of errors in the east direction of the fusion calibration algorithm is improved by 0.33 dB and 1.57 dB compared with that of the LM calibration algorithm based on GPS. The error of the gravity misalignment angle decreases correspondingly, indicating that the calibrated MEMS array has better accuracy in the trajectory tour planned in inertial navigation.

## 5. Conclusions

This paper presents a calibration method to accurately calibrate the fixed error of MEMS arrays by placing the MEMS array in different attitude transitions, using static intervals to calibrate the accelerometer and dynamic intervals to calibrate the gyroscope. The simple experimental procedure also makes the proposed algorithm easy to implement. Using this calibration scheme, a large amount of data can be collected from the MEMS array, further providing the necessary conditions to calculate the optimal solution for the global fixed error parameters of the MEMS array, which can then be calibrated using an adaptive fusion algorithm to estimate the global optimal value. Experiments on real data sets show that the model and fusion calibration algorithms proposed in this topic are feasible, and the method is shown to be more accurate for fusion-calibrated MEMS arrays than for individual MEMS calibrated by the LM-calibration algorithm in static validation, dynamic validation, and navigation validation, with the following key findings:

(1) Building a high-precision static and dynamic screener for M-IMU: for the M-IMU calibration, we do not use high-cost calibration instruments as experimental equipment, rather, on the basis of a redundant consumer-grade MEMS composition array using the original data of the MEMS array multi-position attitude rotation and stationary interval for accurate estimation of the error parameters of the array MEMS, this subject uses ALLAN variance to calculate the stationary state of the initialization time $T_{init}$ and defines the variance of the output data of the MEMS array as a covariate during the period of $T_{init}$ for weighted average. Its weighted average value is the threshold value for judging the static and dynamic of this M-IMU system, which is greater than the threshold value per unit of time as dynamic, and less than the threshold value as static, and this filter can accurately distinguish the different state intervals required for different sensors.
(2) Construction of a calibration parameter optimization algorithm for individual MEMS: For individual MEMS arrays subject to fixed errors, a non-linear calibration factor based on the LM-calibration algorithm is proposed, which optimizes the deterministic error parameters by calculating a non-linear cost function constructed according to the IMU error model and using the LM algorithm to iteratively optimize the cost function in different state intervals. The method is able to guarantee the reliability of the data on the premise of faster convergence; that is, the sensor cost function in multiple targets can quickly converge to the optimal value.
(3) Constructing a fusion calibration algorithm for MEMS arrays: For MEMS arrays affected by the error of autocorrelation and intercorrelation of MEMS*_i_* and MEMS*_j_*, this paper uses the gravity coordinate system of MEMS0 as the reference, and the angular rates of the remaining MEMS sensitive axes are mapped with the reference as the system coordinate system. Each MEMS after coordinate transformation is calibrated with fixed error parameters, and the fixed error parameters of the obtained MEMS arrays are subjected to a fusion algorithm for improving adaptive support based on a priori information. The fusion-calibration algorithm’s support quotes an exponential function that efficiently and accurately quantifies the support of the sensor observations, allowing the MEMS array fixed error parameters to be fused into the error parameters of a “high precision virtual inertial guidance” MEMS0 along the way.
(4) The feasibility of the calibration algorithm is verified in a practical environment from multiple perspectives: the traditional validations of calibration results are: validation of the data set, validation of the variance of the fixed error parameters, and validation of the static output of the sensor. The above-mentioned validation methods are carried out at the system level in an ideal laboratory environment, and the non-linearity of the calibration factor cannot be accurately verified in real-world situations. The dispersion of the fused and calibrated sensor data converges exponentially. The fusion-calibrated M_IMU outdoor navigation trajectory in the real environment also overlaps well with the GPS-planned trajectory [39], and the horizontal orientation error is small. By comparing the calibrated trajectories of different calibration algorithms, it is verified that the proposed fusion calibration algorithm has a high robustness in the real environment.

## Figures and Tables

**Table 1:** Comparison of fusion-calibration algorithms with conventional algorithms.

	Simultaneous Calibration of MEMS Arrays	Duration of Calibration (Min)	Real-Time Calibration	Auxiliary Equipment	Accuracy of Calibration
Multi-position swing	Not applicable	20	Not applicable	YES	Excellence
Multi-rate rotation	Not applicable	20	Not applicable	YES	Excellence
Traditional LM-calibration	Not applicable	5	Applicable	NO	Good
Fusion- calibration	Applicable	7	Applicable	NO	Excellence

**Table 3:** Fixed error parameters for accelerometers and gyroscopes.

	MEMS0	MEMS1	MEMS2	MEMS3	MEMS4	MEMS5	MEMS6	MEMS7	MEMS8	MEMS9
*β_yz_*	0.0156	0.0129	−0.0087	−0.0098	−0.0098	0.0045	0.0073	0.0118	0.0063	−0.0078
*β_zy_*	0.3246	0.1244	0.0501	0.0440	0.0440	−0.0415	−0.0558	−0.1911	−0.1737	−0.0478
*β_xz_*	0.0134	0.0127	0.0098	0.0100	0.0119	0.0100	0.0098	0.0103	0.0135	0.0103
*β_zx_*	−0.1703	−0.1345	0.0116	0.0010	0.0010	−0.0017	0.0119	0.0166	−0.1746	0.0135
*β_xy_*	−0.0201	−0.0201	−0.0201	−0.0201	−0.0201	−0.0201	−0.0201	−0.0201	0.0201	0.0201
*β_yx_*	0.0098	0.0098	0.0098	0.0098	0.0098	0.0098	0.0098	0.0098	−0.0098	0.0098
*α_yz_*	0.4074	0.1613	−0.9850	−0.2787	0.0978	−0.3137	−0.7320	−0.0314	0.0600	0.0963
*α_zy_*	0.3176	−0.0346	−0.0255	2.3920	0.0872	−3.1487	1.2964	0.1114	0.0658	−1.4672
*α_xz_*	0.5530	0.1421	−0.4196	−2.0693	−0.2942	−1.8412	−1.5592	0.0224	0.0534	−1.9963
*α_zx_*	−0.3486	−0.3173	0.6723	−0.1927	−0.1102	0.6250	1.4246	−0.0464	0.1800	0.2984
*α_xy_*	−0.1713	0.1323	0.9803	−0.2584	0.7119	2.1076	0.7412	−0.1229	−0.0744	0.0173
*α_yx_*	0.2140	0.6368	−0.2490	−2.6332	−0.6268	−3.3696	−1.6540	0.1367	0.1282	−1.7084
*S_ax_*	1.1774	0.9793	−1.0370	−0.9737	1.1470	−0.9557	0.9467	0.9770	0.9515	0.9571
*S_ay_*	0.9009	0.9881	−0.9203	1.0206	0.9232	−1.0450	0.9548	1.1411	1.1741	0.9739
*S_az_*	0.9775	1.1296	−1.1527	−1.1377	−0.8155	−1.1217	0.9822	0.9771	−0.9602	1.1010
*S_gx_*	1.4908	1.7746	−2.9116	2.8745	3.0908	−1.3875	−1.4758	0.5557	−1.3609	−0.5601
*S_gy_*	−3.0940	−3.0137	−0.5167	−1.6659	1.3196	3.1842	2.9777	−1.6535	−3.0783	3.0474
*S_gz_*	−0.5662	0.5544	1.6354	0.5374	0.5623	0.5511	0.5471	3.0038	0.5633	0.5968
*b_ax_*	0.0125	0.0013	0.0071	0.0183	−0.0029	−0.0180	0.0006	0.0034	−0.0231	0.0028
*b_ay_*	0.0016	−0.0146	0.0072	−0.0037	−0.0065	0.0013	−0.0264	−0.0123	−0.0154	−0.0235
*b_az_*	0.0078	0.0025	−0.0073	−0.0098	0.0040	−0.0065	0.0151	0.0011	−0.0107	0.0097
*b_gx_*	0.4659	0.2948	0.5430	0.7256	−0.3401	0.0263	0.6740	−0.4590	0.0745	0.6452
*b_gy_*	0.1180	0.4999	0.7233	0.0607	0.3091	−0.1212	0.6842	−0.3417	−0.7949	0.2644
*b_gz_*	−0.3766	−0.1295	0.0336	−0.0817	−0.0485	−0.5892	−0.6529	−0.3511	0.7438	−0.6763

**Table 4:** Calibration factors for MEMS0 accelerometers and gyro-sensitive axes.

Parameters	*X*-Axis	*Y*-Axis	*Z*-Axis
Accelerometers	1.0659	0.9485	1.0356
Gyroscope	1.2683	3.0275	0.5551

**Table 5:** Zero bias of MEMS0 accelerometers and gyroscopes.

Parameters	*X*-Axis	*Y*-Axis	*Z*-Axis
Accelerometers	0.0090	−0.0112	0.0074
Gyroscope	0.4254	0.3912	−0.3682

**Table 6:** Misalignment errors of MEMS0 accelerometer and gyro-sensitive axes.

Parameters		*X*-Axis	*Y*-Axis	*Z*-Axis
Accelerometers	*X*-axis	0.9990	0.0089	−0.0453
	*Y*-axis	0.0112	1.0001	0.0451
	*Z*-axis	0.0201	0.0098	0.9998
Gyroscope	*X*-axis	1.6206	−0.2243	−0.6300
	*Y*-axis	−0.6494	1.5533	−0.1190
	*Z*-axis	−0.3780	−0.6812	1.4497

**Table 7:** MEMS0 accelerometer performance parameter ratio graph.

Axial	*X*-Axis		*Y*-Axis		*Z*-Axis	
Parameters	Average	RMSE	Average	RMSE	Average	RMSE
Raw data	0.0125	2.3559 × 10^−4^	−0.0235	3.7514 × 10^−5^	0.9903	9.2439 × 10^−5^
LM-calibration	0.0028	2.9632 × 10^−6^	−0.0182	5.9399 × 10^−6^	1.0078	3.0600 × 10^−6^
Fusion-calibration	0.0002	5.0428 × 10^−7^	−0.0092	2.7991 × 10^−7^	1.0019	9.4217 × 10^−7^

**Table 8:** MEMS0 gyroscope performance parameter ratio chart.

Axial	*X*-Axis		*Y*-Axis		*Z*-Axis	
Parameters	Average	RMSE	Average	RMSE	Average	RMSE
Raw data	0.6452	2.1370	0.2644	0.0425	−0.3766	0.3137
LM-calibration	0.4659	0.0385	0.2466	0.0054	−0.3341	0.1371
Fusion-calibration	0.2703	0.0021	0.1180	0.0034	−0.3118	0.0105

**Table 9:** Comparison of MEMS0 accelerometer parameters under dynamic conditions.

The Axis of Rotation	MEMSO Accelerometer (m/s^2^)						
	Axial	Raw Data		LM-Calibration		Fusion-Calibration	
	Parameters	Average	RMSE	Average	RMSE	Average	RMSE
*X*-axis	*X*-axis	9.7673	0.1246	9.7674	0.0312	9.8226	0.0081
	*Y*-axis	−0.0858	0.9835	−0.0820	0.2060	−0.0715	5.2899 × 10^−5^
	*Z*-axis	0.7076	1.3006	0.7070	0.4738	−0.0751	6.8966 × 10^−5^
*Y*-axis	*X*-axis	0.0111	2.5050	0.0110	0.4441	−0.0090	6.3637 × 10^−5^
	*Y*-axis	9.6402	1.1647	9.7401	0.5740	9.8221	0.0205
	*Z*-axis	0.3766	2.4213	0.3655	0.3364	−0.0199	1.1143 × 10^−4^
*Z*-axis	*X*-axis	−0.1488	0.6900	−0.1486	0.0206	−0.0005	5.4024 × 10^−5^
	*Y*-axis	0.0138	0.1337	0.0100	0.0028	0.0110	5.4158 × 10^−5^
	*Z*-axis	9.8309	0.6390	9.8306	0.5420	9.8009	0.4667

**Table 10:** Comparison of MEMS0 gyroscope parameters under dynamic conditions.

The Axis of Rotation	MEMSO Gyroscope (dps)						
	Status	Raw Data		LM-Calibration		Fusion-Calibration	
	Parameters	Average	RMSE	Average	RMSE	Average	RMSE
	Stationary	0.0759	0.0966	0.0073	1.1726 × 10^−4^	0.0020	3.5822 × 10^−5^
*X*-axis	Rotation	9.7167	0.0031	9.8170	1.1910 × 10^−4^	9.9348	4.4647 × 10^−5^
	Stationary	0.0210	0.0035	0.0032	1.8668 × 10^−4^	0.0014	1.5160 × 10^−4^
	Stationary	0.4177	2.0450	0.0199	6.9120 × 10^−4^	0.0113	2.1360 × 10^−4^
*Y*-axis	Rotation	9.7987	0.0044	9.8999	1.3235 × 10^−4^	9.9829	1.0088 × 10^−4^
	Stationary	0.0100	0.0847	0.0108	0.0016	0.0019	4.6261 × 10^−4^
	Stationary	0.4534	2.6219	0.0014	8.3999 × 10^−5^	−0.0004	3.6236 × 10^−5^
*Z*-axis	Rotation	9.8063	0.0046	9.9052	3.3486 × 10^−4^	9.9886	3.2120 × 10^−5^
	Stationary	0.1740	0.0048	0.0400	1.7718 × 10^−4^	0.0025	3.4964 × 10^−5^

**Table 11:** Vertical comparison error and horizontal comparison error.

Calibration Algorithms	RMSE in North Direction (m)	RMSE in East Direction (m)	Initial Gravity Misalignment Angle Error (°)
GPS	0	0	0
LM-calibration	1.0637	1.2263	2.56
Fusion-calibration	0.9856	0.8541	0.56
